# The effectiveness of insulin glargine 300 U/mL among type 2 diabetes patients: Analysis of a real‐world data in Israel

**DOI:** 10.1002/edm2.124

**Published:** 2020-03-24

**Authors:** Cheli Melzer Cohen, Tamar Banon, Varda Shalev, Gabriel Chodick

**Affiliations:** ^1^ Maccabi Institute for Research and Innovation Maccabi Healthcare Services Tel Aviv Israel; ^2^ School of Public Health Sackler Faculty of Medicine Tel Aviv University Tel Aviv Israel

**Keywords:** basal insulin, glargine‐300, observational study, type 2 diabetes mellitus

## Abstract

**Aims:**

Randomized controlled trials have shown that insulin glargine 300 U/mL (Gla‐300) has a more stable and prolonged glucose lowering effect among patients with type 2 diabetes (T2DM) compared to insulin glargine 100 U/mL (Gla‐100), resulting in a reduced risk of hypoglycaemia while maintaining a similar efficacy of lowering HbA_1c_. We aimed to investigate if the effectiveness of Gla‐300 is reproducible in real‐world settings.

**Material and methods:**

In this retrospective cohort study, data from a large state‐mandated health organization were used to identify adult T2DM patients who were previously on insulin and initiated Gla‐300 therapy between 6/ 2016 and 12/2017. Changes in HbA_1c_ levels, body weight and insulin dose were calculated from baseline period and over a follow‐up period of 180 days. Documented hypoglycaemia events were also explored.

**Results:**

A total of 1797 patients were included in this study with a mean age of 64.2 (SD = ±11.0y), baseline HbA_1c_ was 8.7 ± 1.6% and 42.5% were females. Among all patients with HbA_1c_ measurement during follow‐up (n = 1508), HbA_1c_ was significantly reduced by −0.6% (95% CI −0.6,−0.5; *P* < .001) from baseline, with a significant reduction in body weight (−0.4 kg; *P *= <.001).

Additionally, a significant (*P* = .04) reduction of 40.5% in patients with hypoglycaemia events was recorded during follow‐up period, from 2.1% (n = 37) at the baseline period to 1.2% (n = 22).

**Conclusions:**

This real‐world study supports evidence from RCTs regarding the effectiveness of Gla‐300 among T2DM patients by improving glycaemic control.

## Graphical abstract

In this real‐world study, the effectiveness and safety among 1797 type 2 diabetes patients were investigated. The article provides evidence that insulin glargine 300 is effective in reducing blood glucose levels without increasing body weight or hypoglycaemic events.

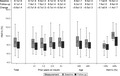

## 1 INTRODUCTION

Type 2 diabetes mellitus (T2DM) is a chronic progressive metabolic condition characterized by insulin resistance and decreased beta‐cell function.^1^ Patients with T2DM suffer from either one or several metabolic abnormalities, such as obesity, increased endogenous glucose output, compromised insulin action and insulin secretion dysfunction.^2^, ^3^, ^4^ This condition places patients at an elevated risk of developing cardiovascular diseases, myocardial infarctions, stoke and elevated blood pressure along with many other potential health impediments.^5^, ^6^

The reduction of functional insulin‐producing beta cells contributes to the development and progression of T2DM.^7^, ^8^ As the nature of this condition is progressive and as beta‐cell activity declines, a glycaemic control and medication regimen should be re‐evaluated routinely and adjusted based on patient characteristics. As T2DM advances, the endogenous insulin may be insufficient to achieve the desired glycaemic control. Based on the American Diabetes Association (ADA) and the European Association for the Study of Diabetes (EASD) recommendations, once the glycaemic control is insufficiently achieved by using other antihyperglycaemic agents, the intensification with insulin should be considered and should not be delayed.^9^, ^10^ Treatment with insulin has the advantage of being highly effective in reducing blood glucose levels as mono‐therapy or in combination with any other agents, especially in severe hyperglycaemia when blood glucose levels reach ≥300 mg/dL or HbA_1c_ ≥ 10%.^11^

Physicians and patients may be reluctant in initiating insulin therapy, where such a delay could increase the risk of long‐term glycaemic complications that may lead to micro and macro‐vascular complications.^12^, ^13^ Although good glycaemic control is related to a lower risk for micro and macro‐vascular complications, fear of the short‐term outcome of hypoglycaemia is associated with delayed insulin initiation and no or low adherence to insulin treatment.^14^

In June 2016, insulin glargine 300 U/mL (Gla‐300; Toujeo ^®^), a longer‐acting second generation basal insulin analogue, was introduced in Israel. This insulin is a new formulation of insulin glargine with a more stable and prolonged glucose lowering effect compared to insulin glargine 100 U/mL (Gla‐100; Lantus^®^).

In the EDITION randomized clinical trial programme, glycaemic control and hypoglycaemia were evaluated by comparing the efficacy and safety of Gla‐300 versus Gla‐100 insulin in patients with T2DM. Gla‐300 was demonstrated its efficiency by lowering HbA_1c_ levels and (significantly) decreasing the risk of hypoglycaemia for nocturnal hypoglycaemia events in patients with T2DM between 6 and 12‐month post‐treatment period in patients previously on basal insulin 15, 16, 17, 18 as well as among insulin naïve patients. 19

Furthermore, in the head‐to‐head BRIGHT RCT, a 24 weeks treatment of Gla‐300 was compared to insulin degludec (IDeg) and demonstrated similar glycaemic control improvement, where Gla‐300 showed lower hypoglycaemic events during titration period.^20^ In the CONCLUDE trial, which was recently presented at the European Association for the Study of Diabetes (EASD), there was no statistical difference in the primary end‐point of number of severe or blood glucose confirmed symptomatic hypoglycaemia episodes between patients who initiated IDeg compared to Gla‐300 initiators.^21^

Recently published real‐world retrospective and prospective evidence studies showed a significant reduction in HbA_1c_ following 6 months of treatment with Gla‐300 insulin.^22^, ^23^ Researchers also recommended administering Gla‐300 insulin over Gla‐100 insulin due to patients' improvements in glycaemic control, low incidence of hypoglycaemic episodes and no weight gain, suggesting that Gla‐300 is a suitable therapy option.

The aims of this study are therefore to investigate the effectiveness and safety of a longer basal insulin, Gla‐300, in a real‐world setting to assist patients, clinicians and health care systems in making informed decisions on effective care and treatment for T2DM patients.

## 2 MATERIALS AND METHODS

### 2.1 Data source

The study utilized de‐identified data from the Maccabi Healthcare Services (MHS) central computerized database, the second largest state‐mandated health provider in Israel, serving more than 2.2 million (25% of the population) members and is a representative sample of the Israeli population. This fully computerized database captures all information on patient interaction (including demographics, inpatient and outpatient visits, diagnoses, procedures, imaging, medications prescriptions and actual dispenses and laboratory measurements). Based on this information, MHS had developed daily updated registries for major chronic diseases, such as hypertension, cardiovascular diseases and diabetes in order to improve disease management and quality of care for MHS members. More than 180 000 patients were accounted for based on medication dispenses, laboratory measurements and diagnoses and defined as having diabetes. Specific inclusion criteria for the diabetes registry are available and are described elsewhere.^24^ As the MHS registry was developed for disease management, it is highly validated.

### 2.2 Study population and design

In this noninterventional retrospective cohort study, we identified adult MHS members (ages > 18 years) who initiated treatment with Gla‐300 from 1 June 2016 until 31 December 2017 (was defined as the index date), included in diabetes registry prior to index date and treated with Gla‐300 for at least 180 days. Additional inclusion criterion was having a baseline HbA_1c_ measurement (last measurement within 180 days prior to index date). Patients who were not on insulin during baseline period were excluded from main analysis and were briefly described in the results. Baseline period was defined as 180 days prior to the index date and follow‐up period was defined as 180 days afterwards. Patients with continuality of less than year in MHS defined as having T1DM or with indication of pregnancy between 9 months prior to index date and 9 months after to end of study were excluded. Discontinuation was defined as the first time having a gap larger than 90 days between two dispenses dates, taking into account cover days.

Reported hypoglycaemic events were based on any diagnoses documented through the MHS database and recorded per outpatient visitation or per hospitalization when necessary.

Approval was obtained from the Institutional Review Board (IRB) and Ethics Committee of MHS for the purposes of accessing and analysing the data. Individual patient informed consent was not required because of the anonymized nature of the patient records.

### 2.3 Study variables

Study population was characterized at baseline and presented by patients' demographics, anthropometric measurements, HbA_1c_ levels, blood lipids, antihyperglycaemic regimen and comorbidities (based on including in MHS registries prior to index date). Anthropometric measurements and laboratory measurements were defined as last measurement during baseline period, while antihyperglycaemic regimen was defined as having at least 1 medication containing the specific ingredient. Cardiovascular disease, hypertension CKD stage and cancer comorbidities were based on our highly validated chronic registries, while revascularization procedure (coronary artery bypass graft and percutaneous coronary intervention) was based on procedures conducted during baseline period.

Differences in documented hypoglycaemia (based on recorded *International Classification of Diseases* version 9 (ICD‐9) codes: 251.0, 251.2, 962.3) during baseline and follow‐up periods were evaluated. Changes in HbA_1c_, weight and BMI were evaluated from baseline (last measurement during baseline period) and follow‐up measurement (closest measurement within 180 ± 90 days after index date). Changes in insulin dosage (both total and basal) were evaluated from baseline period and follow‐up period and during follow‐up period (0‐90 and 91‐180 days after the index date) among patients who were on insulin for at least 180 days prior to index date. The daily insulin dosage was evaluated by using information on actual dispenses, which included data on the amount of insulin dispensed in specific time frames.

### 2.4 Statistical analysis

Descriptive statistics for all baseline characteristics were presented as mean and standard deviation (SD) for continuous variables and as counts and percentages for categorical variables. For continuous variables, statistical analyses were performed by using the t test, or Wilcoxon‐signed rank test, as appropriate. Same procedures were used to compute means and percentages in the various subgroups of patients, as defined in the protocol. Standardized mean difference effect size (*d*) was calculated to assess the magnitude of change between baseline and follow‐up period as follows:

$$d=\text{abs}\left(\frac{\text{Mean}\left(\text{pre}\right)-\text{mean}\left(\text{post}\right)}{\text{SDdiff}}\right)$$

The magnitude of *d* is as follows: 0.01‐ very small, 0.2‐ small, 0.5‐ medium, 0.8‐ large, 1.2‐ very large and 2‐ huge.

Differences in hypoglycaemia were tested by using McNemar's test. A two‐sided P‐value of <.05 was considered as statistically significant.

## 3 RESULTS

A total of 1797 patients were eligible for the study. Baseline characteristics, laboratory measurements, baseline antihyperglycaemic levels and other parameters are presented in Table 1. Briefly, patients who switched to Gla‐300 insulin had a mean (SD) age at index date was 64.2 ± 11.0 years, 42.5% were female, and 77.5% were included in the diabetes registry for more than 10 years. The mean HbA_1c_ at baseline was 8.7 ± 1.6%, mean BMI was 31.9 ± 5.5 kg/m^2^ where 75.0% had hypertension.

**Table 1:** Baseline characteristics of included patients (N = 1797)

Parameter	Category	n (%)
Age (years)	Mean ± SD	64.2 ± 11.0
Sex	Females	764 (42.5)
Time since DM diagnosis	≤2 y	33 (1.8)
	2‐10 y	372 (20.7)
	10+ y	1392 (77.5)
Antihyperglycaemic medications		
Metformin		1162 (64.7)
SU		222 (12.4)
DPP‐4i		404 (22.5)
GLR‐1RA		449 (25.0)
SGLT‐2i		354 (19.7)
Rapid insulin		733 (40.8)
Basal insulin		1725 (96.0)
Premix insulin		128 (7.1)
Other insulin		15 (0.8)
Time (years) on insulin	≤1	114 (6.3)
	1‐2	136 (7.6)
	2‐5	520 (28.9)
	5+	1027 (57.2)
Other antihyperglycaemic medication		309 (17.2)
Number of antihyperglycaemic medications at baseline	0‐1	388 (21.6)
	2	456 (25.4)
	3	559 (31.1)
	4+	394 (21.9)
HbA_1c_ (%); n = 1797	Mean ± SD (Median)	8.7 ± 1.6 (8.4)
LDL (mg/dl); n = 1461	Mean ± SD (Median)	84.2 ± 31.3 (79.6)
HDL (mg/dl); n = 1715	Mean ± SD (Median)	42.1 ± 11.4 (40.0)
non‐HDL (mg/dl); n = 1715	Mean ± SD (Median)	121.4 ± 40.8 (114.0)
Body weight (kg); n = 1792	Mean ± SD (Median)	88.4 ± 17.0 (87.0)
BMI (kg/m^2^); n = 1792	Mean ± SD (Median)	31.9 ± 5.5 (31.0)
Cardiovascular disease		681 (37.9)
Hypertension		1348 (75.0)
CKD stage 3 or worse		558 (31.1)
Cancer		299 (16.6)
Coronary revascularization procedure		387 (21.5)

Abbreviations: BMI: body mass index; CKD: chronic kidney disease; DM: diabetes mellitus; DPP‐4i: Dipeptidyl Peptidase 4 inhibitor; GLP‐1RA: Glucagon‐like peptide 1 receptor agonist; HDL: High‐density lipoprotein; LDL: Low‐density lipoprotein; SD: standard deviation; SGLT‐2i: Sodium‐glucose cotransporter 2 inhibitor; SU: Sulfonylurea.

**Figure 1:**
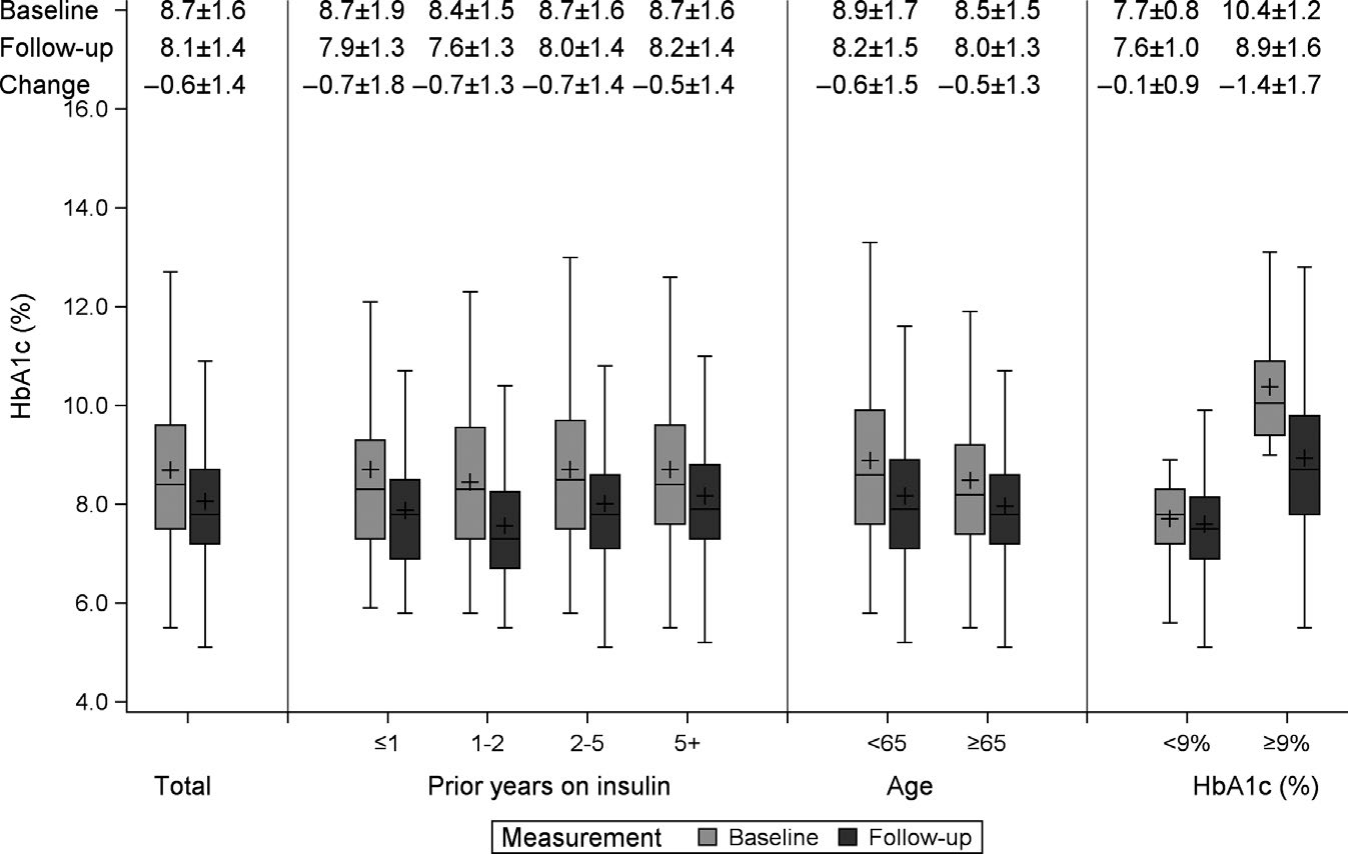
Box‐plot of HbA_1_ **_c_** levels at baseline and during follow‐up period, identifying Mean ± SD for baseline, follow‐up and change at the top of figure. Change in HbA_1_ **_c_** was statistically significant (*P*‐value < .001) in all subgroups. *P*‐value for heterogeneity was 0.018, 0.016 and <0.001 for prior years on insulin, age and baseline HbA_1c_, respectively

For the 1508 patients with the HbA_1c_ measurement during follow‐up, HbA_1c_ was decreased by −0.6% (95% CI: −0.6, −0.5; *P *< .001; *d*: 0.4) from 8.7 ± 1.6 to 8.1 ± 1.4. Higher reduction was observed among patients aged less than 65, patients with higher levels of baseline HbA_1c_ and among patients who were on insulin for less than 5 years (Figure 1).

There were 1206 patients with valid body weight measurement during baseline and during follow‐up. Among them, statistical significance changes in body weight and in BMI were observed (−0.4 (95% CI: −0.6, −0.2; *P* < .001; *d*: 0.1) and −0.1 (95% CI: −0.2, −0.1; *P* < 001; *d*: 0.1), respectively).

During the study follow‐up period, a total of 22 patients reported hypoglycaemic events (12.2 per 1000) compared to 37 patients (20.6 per 1000) during the baseline period, representing a 40.5% reduction (*P* = .039). When stratifying population by time on insulin, trends were similar; however, results were not statistically different. Among patients on insulin for <5 years prior to index date, six patients (7.8 per 1000) were reported with hypoglycaemic events during follow‐up compared to the 11 patients (14.3 per 1000) at baseline (*P* = .225). For those who were on insulin for over 5 years prior, 16 patients (15.6 per 1000) reported hypoglycaemic events at follow‐up in comparison with 26 (25.3 per 1000) at baseline (*P* = .096).

**Figure 2:**
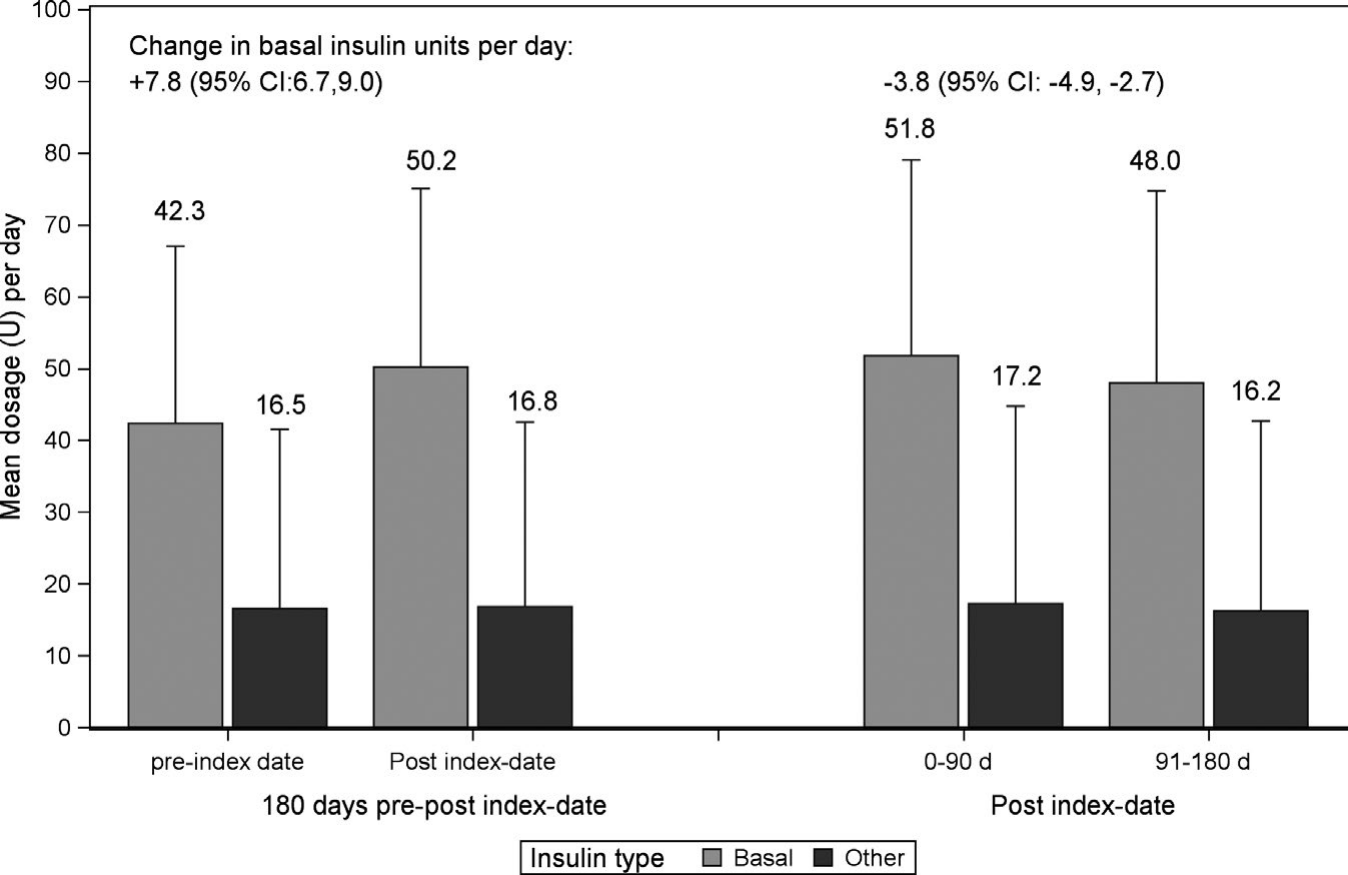
Change in mean (+SD) insulin daily dosage during study period. Change in Insulin was calculated among 1533 patients who were on insulin during all baseline period. *P*‐value < .001 for change in basal insulin (both periods) and *P*‐value = .02 for other insulin during post index date period

Change in insulin dosage was calculated only among patients who were on insulin for at least 180 days prior to index date (n = 1533). Among these patients, the insulin daily dosage was increased during baseline and follow‐up periods by 7.8 (95% CI: 6.7, 9.0) and 0.3 (95% CI: −0.6, 1.1) units for basal and other insulin (mainly fast acting insulin), respectively. During the follow‐up period, both basal and other insulin (mainly fast acting insulin) dosage were decreased by −3.8 (95%CI: −4.9, −2.7) and −1.0 (95% CI: −1.9, −0.2) units per day for basal and other insulin, respectively, starting from the first 90 days of treatment initiation and then following 90 days after (Figure 2).

An additional 607 patients were excluded from the main analysis as they were insulin naïve and therefore were evaluated separately. Their mean (SD) age at index date was 63.3 ± 12.4 years, 42.7% were female, and 57.0% were included in the diabetes registry for more than 10 years. At baseline, HbA_1c_ was 9.6 ± 2.1%, BMI was 30.2 ± 5.5 kg/m^2^ and 67.1% of patients had hypertension. A reduction of −1.7% (95% CI: −1.9, −1.5; *P *< .001) in HbA_1C_ and an increase of 1.1 kg (95% CI: 0.6, 1.6; *P *< .001) in body weight were observed. No reported hypoglycaemia events were reported during baseline and follow‐up periods.

## 4 DISCUSSION

The results of this real‐world data analysis indicate that treatment with Gla‐300 is associated with a significant reduction in both HbA_1c_ levels and in reported hypoglycaemia events, with no increase in patients' body weight.

Adherence to insulin therapy is critical to manage T2DM and to reduce the risk of complications. In an international cross‐sectional web survey of people with T2DM, those who discontinued treatment with basal insulin reported weight gain (48.4%) and hypoglycaemia (25.8%) as the main reason for discontinuation.^25^ Another web‐based survey also suggested that hypoglycaemia can negatively impact many aspects of daily life and that choosing the appropriate treatment is necessarily in order to prevent or control future episodes.^26^

Our study results are in accordance with a recent US study where severe hypoglycaemic events were up to 30% lower for a group patients who switched to Gla‐300 in comparison with first generation basal insulin analogues (Gla‐100 and detemir), where rates were similar with IDeg and Gla‐300 patient groups.^27^ Similarly, results from another parallel group study compared the efficacy and safety of Gla‐100 and Gla‐300 insulin by evaluating nocturnal hypoglycaemic events; researchers found 46% of patients with Gla‐100 had nocturnal episodes in comparison with 36% of patients with Gla‐300 dosages of insulin after a 6‐month treatment period.^17^ The larger reduction of hypoglycaemic events in the Gla‐300 insulin group corresponds with our results and suggests that Gla‐300 may be superior in diminishing glycaemic events, thus increasing the probability for T2DM patients to live more comfortably and securely.

A continued issue of the previously mentioned study was also published where researchers identified both daytime and nocturnal hypoglycaemic episodes with two comparison groups. The results indicated that Gla‐300 was just as effective at Gla‐100 in lowering HbA_1c_ levels, it was associated with a lower risk of glycaemic events, and it demonstrated no weight gain (participants in the Gla‐100 group had an average of 0.66 kg gain). 18 In addition, a comparative study on the effectiveness of T2DM patients switching from Gla‐100 or detemir to Gla‐300 treatment compared to those switching to an insulin IDeg treatment suggested that Gla‐300 had a lower inpatient/emergency department hypoglycaemia event rate at follow‐up, yet both types of insulin had similar glycaemic control improvements.^28^ In our study, we observed a small but statistically significant decrease in body weight and in BMI, where the dosage of Gla‐300 insulin was increased, which reinforces the safety characteristic for using larger amount of insulin. This dosage increase was, however, accompanied with a significant reduction in HbA_1c_ levels from the baseline period compared to the follow‐up period.

In comparison with these results, a similar retrospective observational study suggested that patients switching to Gla‐300 from different basal insulin had significant lower daily doses of basal insulin, fewer hypoglycaemic events and significantly lowered HbA_1c_ levels.^29^

The study enjoys several strengths including a relatively large study population, no loss to follow‐up, cohort study design and a systematic data collection including data on repeated lab results, clinical history and purchased medications. Some limitations should be also discussed. In the current analysis, we did not have a comparison group; therefore, we are unable to assess if the changes in HbA_1c_, body weight and reported hypoglycaemia events observed during follow‐up period were solely due to the larger Gla‐300 dosages or other concurrent factors. Another limitation in our study is lower amounts of hypoglycaemic events were observed compared to other research. This occurrence may be related to the fact that MHS data may not have a complete representation of all hypoglycaemic events that occurred; some events may not have been accounted for by patient reports.

A third limitation is therefore a potential surveillance bias where patients put on new insulin are more likely to attend clinician offices and report hypoglycaemic episodes. However, such bias would have resulted in an increase in hypoglycaemia rate during follow‐up period. Furthermore, our last limitation is the calculated dosage form of insulin based on purchases; we do not know the exact amount of insulin injected to the patients, but there was access to the amount dispensed at baseline and at follow‐up periods. From this information, the daily dosage of insulin was calculated.

In conclusion, this real‐world study corroborates the findings of previous RCTs demonstrating the effectiveness and safety of Gla‐300 insulin among patients with T2DM.

## CONFLICT OF INTEREST

This work was funded by Sanofi. Cheli Melzer Cohen, Tamar Banon, Varda Shalev and Gabriel Chodick have nothing to declare.

## AUTHOR CONTRIBUTIONS

CMC, TB, VS and GC contributed to the development of the study concept, study design and interpretation of the data. CMC contributed to data collection and analysis. CMC, TB and GC contributed to writing the manuscript. VS contributed to the critical review and revision of the manuscript. All authors approved the final version and agreed to be accountable for all aspects of the work.

## Data Availability

Data sharing is not applicable in this research article due to privacy matters, and therefore, it was not approved by the IRB.
